# Cancer Stem Cell Plasticity – A Deadly Deal

**DOI:** 10.3389/fmolb.2020.00079

**Published:** 2020-04-30

**Authors:** Archana P. Thankamony, Kritika Saxena, Reshma Murali, Mohit Kumar Jolly, Radhika Nair

**Affiliations:** ^1^Cancer Research Program, Rajiv Gandhi Centre for Biotechnology, Thiruvananthapuram, India; ^2^Manipal Academy of Higher Education (MAHE), Manipal, India; ^3^Centre for BioSystems Science and Engineering, Indian Institute of Science, Bengaluru, India

**Keywords:** cancer stem cells, plasticity, epithelial-mesenchymal transition, metastasis, microenvironment, metabolic plasticity

## Abstract

Intratumoral heterogeneity is a major ongoing challenge in the effective therapeutic targeting of cancer. Accumulating evidence suggests that a fraction of cells within a tumor termed Cancer Stem Cells (CSCs) are primarily responsible for this diversity resulting in therapeutic resistance and metastasis. Adding to this complexity, recent studies have shown that there can be different subpopulations of CSCs with varying biochemical and biophysical traits resulting in varied dissemination and drug-resistance potential. Moreover, cancer cells can exhibit a high level of plasticity or the ability to dynamically switch between CSC and non-CSC states or among different subsets of CSCs. In addition, CSCs also display extensive metabolic plasticity. The molecular mechanisms underlying these different interconnected axes of plasticity has been under extensive investigation and the trans-differentiation process of Epithelial to Mesenchymal transition (EMT) has been identified as a major contributing factor. Besides genetic and epigenetic factors, CSC plasticity is also shaped by non-cell-autonomous effects such as the tumor microenvironment (TME). In this review, we discuss the latest developments in decoding mechanisms and implications of CSC plasticity in tumor progression at biochemical and biophysical levels, and the latest *in silico* approaches being taken for characterizing cancer cell plasticity. These efforts can help improve existing therapeutic approaches by taking into consideration the contribution of cellular plasticity/heterogeneity in enabling drug resistance.

## Introduction

Heterogeneity in cancer biology has long been recognized and exploited in the clinical management of the disease (Rich, 2016). Intertumoral heterogeneity within breast cancer patients, for example, exhibiting different molecular subtypes based on immunohistochemical markers like Estrogen Receptor (ER) or Her2, has been the basis of successful targeted therapeutic approaches (Turashvili and Brogi, 2017; Januskeviciene and Petrikaite, 2019). The inbuilt cellular variation within a tumor has been shown to be an important driver for the emergence of therapy resistant clones which ultimately lead to recurrence and spread of the cancer cells resulting in patient mortality (Somasundaram et al., 2012; Dagogo-Jack and Shaw, 2018; Akgul et al., 2019).

**FIGURE 1:**
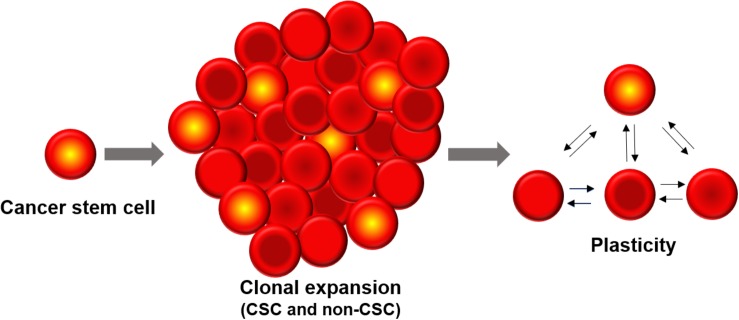
Cancer stem cells (CSCs) constitute a minor sub-population of tumor mass. Phenotypic plasticity can enable CSCs and non-CSCs to interconvert among one another, depending on cell-intrinsic (e.g., epigenetic) and cell-extrinsic (e.g., tumor microenvironment) features.

The development of intratumoral diversity in tumor cells has been widely attributed to two contrasting processes (Shackleton et al., 2009; Prasetyanti and Medema, 2017). The clonal evolution theory takes into account the intrinsic differences between all cells based on genetic and epigenetic programs as well as the influence of the tumor microenvironment (TME). The fitter clones are selected for and contribute to the diversity of the tumor cell population (McGranahan and Swanton, 2017). The second model – Cancer Stem Cell (CSC) model – proposes that there are a subset of cells (termed CSCs) which are predisposed to drive the tumor progression, metastatic and therapeutic resistance of the entire tumor. In this hierarchical model, CSCs can differentiate into less self-renewing populations of non-CSCs which form the bulk of the tumor, in an analogous fashion to stem cell development (Vermeulen et al., 2012). More recently, the ability of cells to switch states via different programs such as Epithelial-Mesenchymal Transition (EMT) has given rise to the concept that non-CSCs can also convert to being a CSC. Thus, CSCs need not be always *a priori* defined; rather stemness can be thought of as a cell state can that be reversibly gained or lost. In other words, cellular plasticity can allow CSCs and non-CSCs to switch among one another (Chaffer et al., 2011; Marjanovic et al., 2013; Gupta et al., 2019). Moreover, different subsets of CSCs can lie on various points on the epithelial-mesenchymal axis and can possibly interconvert (Liu et al., 2014; Bocci et al., 2018; Bocci et al., 2019). Therefore, clonal evolution and CSC models are not necessarily mutually exclusive and the plasticity model ushers in more complexity to the manner in which heterogeneous cell populations can possibly arise within a tumor (Cabrera et al., 2015; Figure 1).

A direct consequence of interconverting or plastic cellular populations in a tumor is the rise of drug resistant and/or metastatic cells which are ultimately responsible for the mortality associated with cancer (Biddle et al., 2016; Doherty et al., 2016). The need of the hour is hence to understand the molecular underpinnings for CSC plasticity and to decode the impact of bidirectional nature of CSC plasticity on the clinical management of the disease.

## CSC Heterogeneity and Plasticity in Tumor Progression

The concept that CSCs are dynamic populations and can undergo spontaneous state transitions has been strengthened by various studies (Chaffer et al., 2011, 2013; Gupta et al., 2011). In the study done by Chaffer et al. (2011), using basal-like breast cancer cells, non-stem cells were shown to spontaneously switch to stem-like cells *in vitro* and *in vivo*; this plasticity was later found to be regulated by ZEB1 (Chaffer et al., 2013) – a key regulator of EMT (Jia et al., 2017). CSC heterogeneity and plasticity has been observed in different cancers. Just like their non-cancerous counterparts, identification of CSCs has been mainly based on the expression of cell surface markers (Chen W. et al., 2016). However, even within a single tumor type, different markers can identify distinct CSCs which are phenotypically distinct and could vary from patient to patient depending on the genetic make-up of the tumor (Tang, 2012). For instance, in glioblastoma, multiple markers like CD133, CD44, A2B5, SSEA have been utilized for identifying the stem cell populations (Singh et al., 2004; Ogden et al., 2008; Son et al., 2009). However, the use of CD133 marker is controversial as CD133^–^ cells have also been shown to form tumors in glioma and CD133^+^ cells could be derived from CD133^–^ cells *in vivo*, implying the underlying plasticity (Wang et al., 2008). A recent study by Dirkse et al. (2019) found that in glioblastoma, the cell-membrane associated CSC markers such as CD133, A2B5, SSEA, and CD15 does not represent a clonal entity but a plastic state which can be adapted by most of the cells in response to varying conditions in the microenvironment. They also proposed that the enhanced tumorigenic potential of CSC-like state is a result of faster adaptation of the cells to the microenvironment.

The evidence for plasticity of CSC states comes from melanoma as well. A slow cycling population of melanoma CSC-like cells were identified using H3K4 demethylase JARID1B as a biomarker (Roesch et al., 2010). Intriguingly, the expression of this marker was dynamically regulated and JARID1B-negative cells could re-express the marker, thus indicating the dynamic nature of the stemness trait. Another seminal study done on melanoma supports the phenotypic plasticity model of CSCs. In this study, phenotypically distinct melanoma cells were shown to undergo reversible phenotypic changes *in vivo* and recapitulate the original tumor (Quintana et al., 2010). In breast cancer, different subsets of CSCs were identified based on ALDH1, CD44, and CD24; and the two subpopulations (epithelial-like ALDH1+, mesenchymal like CD44^+^/CD24^–^) were shown to be capable of inter converting among themselves as well as give rise to non-CSCs (Liu et al., 2014). Moreover, in breast cancer, CSCs and non-CSCs were shown to exhibit dynamic equilibrium maintained by cytokine-mediated crosstalk among these distinct populations (Iliopoulos et al., 2011). These results suggest that at least in some cancers, phenotypic plasticity is reversible and does not necessarily depend on genetic alterations (Jolly et al., 2018a).

Another compelling evidence for CSC plasticity in tumor progression comes from studies on colorectal cancer. LGR5, a Wnt target gene, is used as a marker for colorectal CSCs. Kobayashi et al. (2012) has established human colon cancer cell lines that express LGR5 and possess CSC properties. However, treatment with an anticancer drug resulted in the conversion of the LGR5^+^ cells into LGR5^–^ cells; the absence of drug drove the transition back from LGR5^–^ to LGR5^+^ cells, suggesting the inherent plasticity. Both of these cell types could reconstitute the tumor *in vivo*. Consistently, targeted ablation of Lgr5^+^ CSCs did not lead to tumor regression *in vivo* as the Lgr5^–^ cells could give rise to Lgr5^+^ cells and sustained the tumor growth. But interestingly, the Lgr5^–^ cells could not form liver metastases (de Sousa e Melo et al., 2017), suggesting that the contribution of CSCs in primary tumor formation and that in metastatic settings may be different. However, contrary to these results, a very recent study has shown that majority of the colorectal cancer metastases were seeded by Lgr5^–^ cells. Interestingly, these cells could re-establish cellular hierarchy by giving rise to Lgr5^+^ cells and thereby reinforcing the concept of plasticity (Fumagalli et al., 2020). Therefore, the ability of CSCs and non-CSCs to switch among one another seems crucial both for the primary tumor and metastatic growth. More recently, some markers for metastatic CSCs have been identified across cancers (Celia-Terrassa and Jolly, 2019).

CSC plasticity has also been observed alongside vasculogenic mimicry (VM) – a hallmark process of cancer cell plasticity in which cancer cells transdifferentiate and acquire endothelial cell like characteristics (Fernandez-Cortes et al., 2019). In triple negative breast cancer, a CD133^+^ cell population with CSC-like traits was found to show the ability to form tube-like structures (Liu et al., 2013). In renal cell carcinoma, using immunohistochemistry analysis of patient samples, the expression of stem cell like markers CD133 and CD44 was found to correlate with VM and high CSC marker expression and VM correlated with poor survival (Zhang et al., 2013). Thus, CSCs may not only interconvert among their sub-groups, but also give rise to different kinds of non-CSC differentiated cells.

## Mechanisms Controlling CSC Plasticity

CSC plasticity is controlled by both cell-intrinsic and cell-extrinsic factors (Poli et al., 2018). Several studies have implied the importance of key transcription factors such as OCT3/4, SOX2, NANOG and KLF4 in modulating the generation of CSCs and regulation of cellular plasticity (Gu et al., 2007; Ben-Porath et al., 2008; Liu et al., 2013; Eun et al., 2017). For example, the introduction of OCT3/4, NANOG and KLF4 retrovirally into human colon cancer cells resulted in enhanced CSC properties and the xenografts of these cells actually resembled the original human tumor tissue (Oshima et al., 2014). Similarly, in glioblastoma, Suva et al. (2014) identified a core set of neurodevelopmental transcription factors (POU3F2, SOX2, SALL2, and OLIG2) that were sufficient to reprogram differentiated glioblastoma cells to CSCs. Tumor suppressor transcription factors like p53, pTEN has also been associated with CSC plasticity (Cabrera et al., 2015; da Silva-Diz et al., 2018; Santoro et al., 2019). Loss of p53 lead to increased expression of Nestin and enable the dedifferentiation of hepatocytes and thereby contributes to cellular plasticity in liver carcinogenesis (Tschaharganeh et al., 2014). Similarly, combined loss of p53/pTEN in clonal prostate epithelial cells caused transformation of multipotent progenitors and lead to epithelial to mesenchymal transition (EMT) (Martin et al., 2011). Moreover, genetic mutations of oncogene like KRAS and tumor suppressor like APC is also linked to the generation of stem-like cells (Easwaran et al., 2014).

Many studies have pointed out various mechanisms of epigenetic regulation such as bivalent chromatin state, DNA methylation, histone modifications in mediating CSC plasticity (Poli et al., 2018). For example, in basal like breast cancer cells, Chaffer and colleagues observed that ZEB1 promoter of non-CSCs is maintained in a bivalent configuration and in response to TGFβ, the chromatin switches to an active state leading to the transcription of ZEB1, consequently converting non-CSCs to CSCs (Chaffer et al., 2013). On the other hand, loss of function of HOXC8, a homeobox gene, in non-tumorigenic mammary epithelial cells due to its promoter DNA hypermethylation has been shown to be associated with CSC pool expansion, increased self-renewal and a transformed phenotype (Shah et al., 2017). A histone modifier, enhancer of zeste homolog 2 (EZH2) is a core member of polycomb repressor complex 2 (PRC2) and mediates transcriptional repression of target genes via the trimethylation of lysine 27 of histone 3 (H3K27me3) (Gan et al., 2018). EZH2 is upregulated in many cancers and its enhanced expression is associated with invasion, migration and stemness (Yamaguchi and Hung, 2014). In breast cancer, overexpression of EZH2 can increase mammosphere formation and self-renewal ability in CSCs (Chang et al., 2011; Wen et al., 2017). In glioblastoma, loss of H3K27me3 can lead to aberrant activation of Wnt pathway which is required for tumorigenicity and CSC maintenance (Rheinbay et al., 2013). On the other hand, in pediatric glioblastomas, the mutations in histone variants H3.1 and H3.3 results in reduced activity of EZH2 and consequently reprograms toward a stem cell-like state (Lewis et al., 2013). These observations suggest that CSCs are capable of exploiting the reversible nature of epigenetic modifications to achieve their plastic nature (Wainwright and Scaffidi, 2017). However, this reversibility also putatively offers an attractive opportunity that needs to be harnessed for therapeutic targeting.

## CSC Plasticity and EMT

EMT is a reversible, dynamic process which is critical during embryonic development and also aberrantly activated during various pathological processes like wound healing, fibrosis and cancer progression (Kalluri and Weinberg, 2009; Jolly et al., 2018d). EMT is characterized by the loss of apico-basal polarity, rearrangements in the cytoskeleton and the acquisition of mesenchymal gene expression signature (Kalluri and Weinberg, 2009). The activation of EMT program is associated with the acquisition of stem like characteristics and has been implicated in different cancers (Mani et al., 2008; Shibue and Weinberg, 2017; Singla et al., 2018; Dongre and Weinberg, 2019). Initial reports suggested that activation of an EMT program endowed cells with traits similar to CSCs, such as enhanced colony formation *in vitro* and enhanced tumorigenesis *in vivo* (Shibue and Weinberg, 2017). Recent studies have, however, presented a more nuanced understanding of the interconnection between EMT and CSCs. Cells that undergo a more extreme version of EMT can lose the stemness gained during the initiation of EMT; thus, cells in a hybrid epithelial/mesenchymal phenotype are much more likely to be stem-like as compared to those on either end of the spectrum – pure epithelial or pure mesenchymal (Bierie et al., 2017; Jolly et al., 2018b). A recent study by Kroger et al. has found that the acquisition of a hybrid phenotype is a critical for the maintenance of tumorigenicity of basal breast cancer cells. Based on CD104/CD44 cell surface antigen expression and by regulating the expression of transcription factors like Zeb1 and Snail, they isolated highly tumorigenic cell population residing stably in a hybrid E/M state. This hybrid E/M cell population showed enhanced stemness which was mediated by increased expression of Snail and Wnt signaling pathway (Kroger et al., 2019). Another interesting study by Pastushenko et al. (2018) looked at the spectrum of EMT states that exist in a tumor rather than the binary fixed state that was accepted for long. The hybrid E/M tumor cells were associated with differences in their transcriptional and epigenetic programs, metastatic potential and also the location within a tumor (Pastushenko et al., 2018). It would be interesting to further understand whether these different hybrid states also respond differently to cues like chemotherapeutic treatment leading to resistance and ultimately relapse in cancer patients.

## The Effect of the Tumor Microenvironment on CSC Plasticity

Besides juxtacrine crosstalk among cancer cells and stromal cells, there are factors secreted by the different cell types that form complex interacting networks in a TME (Swartz et al., 2012; Quail and Joyce, 2013; Peltanova et al., 2019). Accumulating evidence suggests that such crosstalk can modulate stem-like behavior and phenotypic plasticity of cancer cells (Pattabiraman and Weinberg, 2014; Cabrera et al., 2015; Prasetyanti and Medema, 2017). Cancer associated fibroblasts (CAFs) are a major component of the TME and play a pivotal role in various aspects of tumor progression (Kwa et al., 2019). CAFs were found to modulate the CSC plasticity in hepatocellular carcinoma through c-Met/FRA1/HEY1 signaling (Lau et al., 2016), in pancreatic adenocarcinoma through FAK signaling (Begum et al., 2019) and in lung cancer by IGF-II/IGF1R signaling pathway (Chen et al., 2014). In a recent study, the extent of intracellular Notch1 signaling in mesenchymal stem cell-derived dermal fibroblasts was found to determine the ability of these cells to regulate melanoma aggressiveness, stemness and phenotypic plasticity (Du et al., 2019). Another key component in TME is the immune system which plays a crucial role in regulating CSC plasticity. In response to chemotherapy, macrophages can secrete factor like Oncostatin-M (OSM), an IL-6 family cytokine which in turn can activate the dedifferentiation of triple negative breast cancer cells into aggressive stem cells (Doherty et al., 2019) and this activation could be mediated through co-operative STAT3/SMAD3 signaling (Junk et al., 2017). OSM can also be secreted by cancer associated adipocytes which can also promote stemness (Wolfson et al., 2015). Similarly, a crosstalk between macrophages of various polarizations (M1, M2) can alter the composition of tumor cells in terms of epithelial vs. mesenchymal populations, thus modulating stemness (Li et al., 2019).

The physical and chemical composition of the microenvironment such as acidic pH, low oxygen and nutrient availability, rigidity and porosity of the ECM can also play an important role in regulating the cancer stem cell behavior (Hjelmeland et al., 2011; Nallanthighal et al., 2019; Prager et al., 2019). A classic example would be hypoxia which is a hallmark of tumor progression in solid tumors and is associated with metastasis, therapeutic resistance and poor survival (Lequeux et al., 2019). A hypoxic microenvironment is known to regulate various aspects of malignant progression including cellular plasticity. In Glioblastoma, hypoxia was found to promote self-renewal in non-stem cells by upregulating important factors like OCT4, NANOG, and cMYC (Heddleston et al., 2009). Also, the hypoxic microenvironment can select the fate of breast cancer stem cells (CSCs) *in vivo* (Kim et al., 2018). Using flow cytometry, hypoxic and non-hypoxic breast cancer cells were isolated from hypoxia sensing xenografts of MDA-MB-231 and MCF7 breast cancer cell lines. Hypoxic tumor cells showed enhanced CSC characteristics compared to non-hypoxic cells which is attributed to the PI3K/AKT signaling. Interestingly, this differential cell fate was observed only in tumor cells isolated from hypoxic TME *in vivo* and not in tumor cells treated by hypoxia *in vitro* alone (Kim et al., 2018).

**FIGURE 2:**
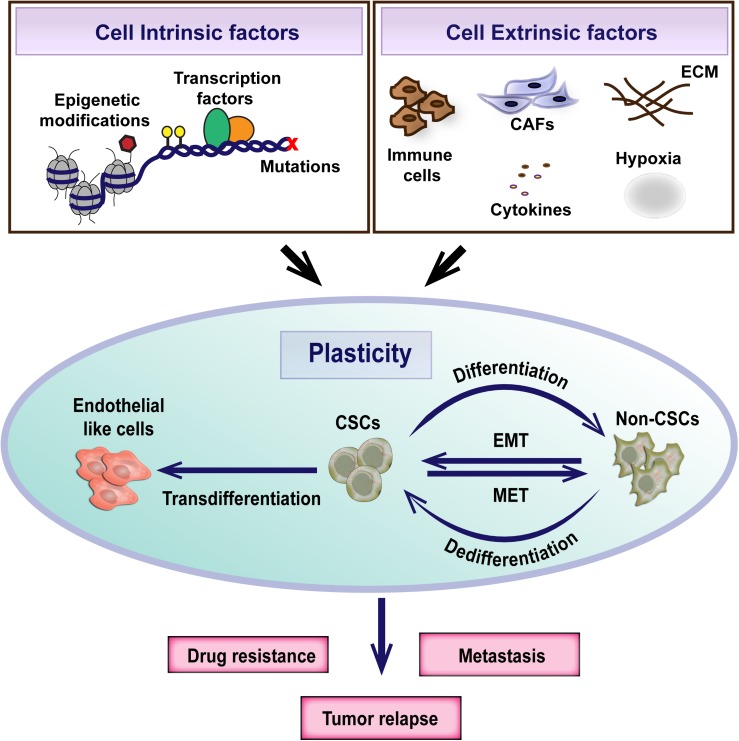
Cancer stem cell plasticity is the ability to dynamically switch between CSC and non-CSC states. It is a complex process regulated by both cell intrinsic and extrinsic factors. Plasticity plays an important role in the evolution of therapeutic resistance, tumor relapse and metastasis.

These studies underscore the importance of the microenvironment in sculpting intra-tumoral heterogeneity and CSC plasticity and highlight the need to better understand the tumor-microenvironment crosstalk for the development of effective therapeutic strategies (Figure 2). However, it is still controversial whether the CSC heterogeneity arises as a consequence of the microenvironment exerted selection pressure or whether plasticity is an intrinsic, default feature of the cancer cells that enable to adapt to varying cues from the microenvironment (Poli et al., 2018; Dirkse et al., 2019). Recent evidence from the study on glioblastoma suggests that intrinsic plasticity of tumor cells enables them to stochastically transition between different states defined by distinct expression of cancer stem cell markers and adapt to the microenvironment. Although all cell subpopulations are capable of phenotypic adjustment, they vary in their speed of adaptation (Dirkse et al., 2019).

## Biochemical Characterization of Cancer Stem Cells and Their Subsets

**FIGURE 3:**
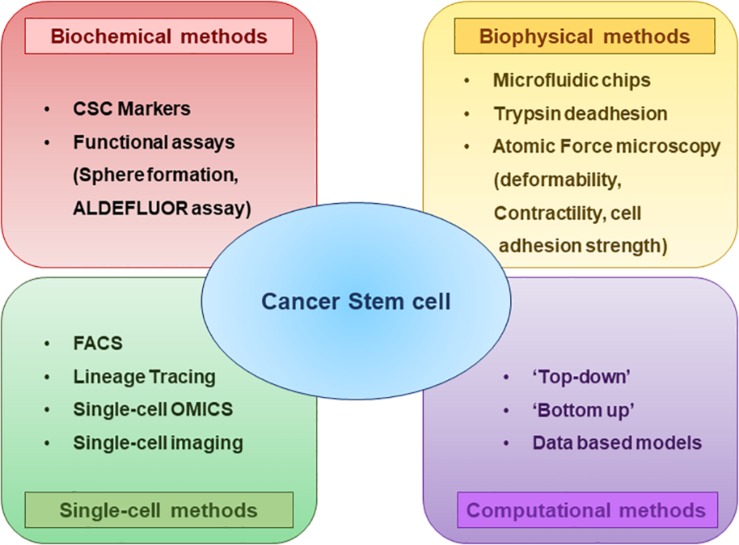
Methods to characterize CSCs and their subsets at a glance. Biochemical and biophysical characteristics of the CSCs can be strikingly different and this diversity can be understood by using multiple assays. Analyzing the properties of CSCs at Single-cell resolution enables to better comprehend the CSC plasticity. Different computational and mathematical models are also being used which helps to gain insights regarding the CSC diversity and plasticity.

CSC plasticity can be instigated by various components in the microenvironment such as the secretion of cytokines and chemokines, communication with different stromal cell types and extracellular matrix and hypoxia (Agliano et al., 2017). Consequent activation of transcription factors and/or epigenetic modifications have been shown to mediate this interconversion (Cabrera et al., 2015). To understand the biology of CSC plasticity and the mechanisms underlying their functional phenotype with the aim of developing efficient treatment strategies, an essential requirement is the characterization and methods to selectively isolate the plastic CSC population from bulk tumors (Figure 3).

One of the serious and longstanding challenges in studying CSCs is the determination of appropriate methodology for the isolation and characterization of CSCs (Agliano et al., 2017). One of the most widely applied method to identify CSCs is to sort the cells based on the expression of cell surface markers such as CD44, CD133, CD24, CD26, EPCAM, CD166 (Jaggupilli and Elkord, 2012; Chen et al., 2013) or based on enzymatic activity of intracellular proteins like ALDH1 (Pattabiraman and Weinberg, 2014; Table 1). However, even these markers are not universally expressed on all CSCs, limiting their use in few cancers (Jaggupilli and Elkord, 2012). To overcome this limitation, more than one markers are used together in several cancers (Agliano et al., 2017). Although multiple markers have been described, the lack of reliable and accurate markers remains to be a stumbling block in the identification of CSCs. Moreover, recent single cell transcriptome analyses revealed that many CSC markers could be co-expressed by a single cell at the same time (Patel et al., 2014; Eun et al., 2017; Table 2) and the expression of CSC markers could vary *in vivo* as a consequence of plasticity and adaptation to the microenvironment (Dirkse et al., 2019). These observations clearly highlight the heterogeneity of CSCs and inefficiency of the markers currently in use in distinguishing CSCs and non-CSCs. Therefore, combining marker-based isolation strategies with functional assays such as *in vitro* clonogenic and *in vivo* limiting dilution xenotransplantation assays are of paramount importance to validate the stemness trait of the cells (Dirkse et al., 2019; Prager et al., 2019; Table 2).

**TABLE 1:** Commonly used markers for the isolation of cancer stem cells.

Cancer type	CSC markers	References
Breast	CD44, CD24, EPCAM, CD133, ALDH	Al-Hajj et al., 2003; Qiu et al., 2019
Glioblastoma	CD133, CD15, CD44, A2B5	Singh et al., 2004; Dirkse et al., 2019
Head and Neck	CD44, CD133, CD98, ALDH, Side population	Prince et al., 2007; Peitzsch et al., 2019
Lung	CD44, CD133, ALDH, CD90	Leung et al., 2010; Maiuthed et al., 2018
Colorectal	CD44, CD24, CD133, CD166, ALDH, EPCAM	Ricci-Vitiani et al., 2007; Zhou et al., 2018
Gastric	CD44, CD24, CD133, LGR5, CD90, CD71	Yang et al., 2007; Bekaii-Saab and El-Rayes, 2017
Pancreatic	CD44, CD24, CD133, ESA, DCLK1, ABCB1	Li et al., 2007; Di Carlo et al., 2018
Hepatocellular	CD44, CD133, CD13, CD45, CD90, EPCAM	Terris et al., 2010; Wang and Sun, 2018
Renal	CD105, CD133, ALDH1	Bussolati et al., 2008; Peired et al., 2016
Ovarian	CD44, CD24, CD117, EPCAM, ABCB1, ABCB2	Zhang et al., 2008; Roy and Cowden Dahl, 2018
Endometrial	CD44, CD117, CD55, CD133	Giannone et al., 2019
Prostate	CD133, CD44, α2β1, ABCG2, ALDH	Collins et al., 2005; Skvortsov et al., 2018
Melanoma	CD133, ALDH, CD271, ABCG2, JARID1B, CD20	Fang et al., 2005; Kumar et al., 2017
Leukemia	CD34, CD38, CD123, CD47, CD96	Lapidot et al., 1994; Wang et al., 2017

**TABLE 2:** Biochemical and biophysical methods to characterize the CSCs and their subsets.

Method	Experiment	Cell-line/Cancer type	Biochemical/Biophysical property	Scale	References
Single-cell RNA sequencing	*In vitro*	Primary glioblastoma cells	CD133	Single cell	Patel et al., 2014
Multi-color flow cytometry	*In vitro*	Glioblastoma tissue isolated from PDX	CD195, CD15, CD95,CD133,A2B5, CD24,CD29, CD44,CD90,CD56	Single cell	Dirkse et al., 2019
Fluorescence activated cell sorting, spheroid assay, RT PCR	*In vitro*	MCF-7, MDA-MB-231, MDA-MB-453	CD44, CD24, Oct4, Nanog and Klf4	Single cell	Srinivasan et al., 2017
Trypsin de-adhesion assay, atomic force microscopy, collagen degradation assay	*In vitro*	MCF-7, MDA-MB-231, MDA-MB-453	ROCK pathway, cell contractility, stiffness, ECM remodeling	Single cell	Srinivasan et al., 2017
Microfluidics method with mechanical separation chip	*In vitro*	MDA-MB-436, MCF-7, SUM149	Deformability, stiffness	Single cell	Zhang et al., 2012
Atomic force microscopy (AFM)	*In vitro*	Murine ovarian surface epithelial (MOSE) cell line	Stiffness	Single cell	Babahosseini et al., 2014
*In vitro* transmigration assay, F-actin staining	Both	tfRFP B16 cells, zebra fish	CDC42, SOX2, deformability	Population	Chen W. et al., 2016
Microfluidic cytometry (MC) chip	*In vitro*	MCF-7, MCF-10A, MDA-MB–231, SUM 149, SUM 159	Cell stiffness and cell-surface frictional property	Single cell	Liu et al., 2015
Microfluidics method	*In vitro*	SUM-149 and SUM-159	Cell adhesion property	Single cell	Zhang et al., 2015
ALDEFLOUR assay, microfluidics method, PDMS micropost array	*In vitro*	SUM149	ALDH, deformability, adhesion strength, contractility, stiffness	Single cell	Chen et al., 2019
Intra-vital lineage tracing	*In vivo*	MMTV-PyMT mouse models of mammary tumor	Cell lineage	Population	Zomer et al., 2013
Lineage tracing, transcriptomic analysis	*In vivo*	Notch1 transgenic mouse models	Cell lineage, Notch1, Lgr5	Population	Mourao et al., 2019
Single-cell RNA sequencing	*In vitro*	Patient-derived primary oral squamous cell carcinomas (OSCC) cell lines	Single cell expression data- biomolecular and epigenetic markers	Single cell	Sharma et al., 2018
Single cell gene expression profiling combined with functional characterization	*In vitro*	ER^+^, ER^–^ breast cancer cell lines	Markers of differentiation, EMT, proliferation, stemness, pluripotency	Single cell	Akrap et al., 2016
Single cell RNA sequencing combined with mammosphere formation assay and label-retention assay	*In vitro*	MDA-MB-231	Markers involved in cell-cycle regulation, stem-cell properties and differentiation	Single cell	Jonasson et al., 2019
High-throughput automated single cell imaging analysis (HASCIA)	*In vitro*	Glioblastoma (GBM) CSCs	CD133, SOX2, pSTAT3,EGFR	Single cell	Chumakova et al., 2019

## Biophysical Characterization of CSCs and Their Subsets

The properties of stem cells such as self-renewal and multipotency can be governed by intra-cellular and extracellular components constituting the stem cell niche (Morrison and Spradling, 2008). Similar to stem cells, properties of CSCs can be regulated by the TME to enhance their metastatic and tumor initiation capabilities (Aponte and Caicedo, 2017). CSCs, on the other hand, can also remodel ECM more strongly as compared to bulk cancer population (Srinivasan et al., 2017); thereby setting a complex feedback loop among the CSC and its niche. Biochemical constituents of such loops have been well-characterized earlier (Korkaya et al., 2011). Recent evidence suggests how biophysical cues such as matrix stiffness, cell contractility and cell-matrix adhesion strengths can regulate the tumor-initiating properties of CSCs. For instance, blocking ROCK (Rho-associated protein kinase) can inhibit cell contractility and invasion potential of breast CSCs (Srinivasan et al., 2017). At a biophysical level, TME is often characterized by increased stiffness due to ECM remodeling, increased compressive stress due to confined growth, enhanced interstitial pressure and an increased interstitial fluid flow (Zanotelli et al., 2018). Extrinsic mechanical forces exerted by ECM constituents can trigger biochemical changes inside cells such as cytoskeleton rearrangement, and changes in gene expression, protein-protein interactions and enzyme modifications, thus converging on various mechano-transduction and mechano-chemical axes (Ogden et al., 2008; Broders-Bondon et al., 2018; Roy Choudhury et al., 2019).

Cancer cells display variations in response to extrinsic biomechanical stimuli, leading to heterogeneity in their biophysical properties, which influences the overall nature of cells such as stemness and differentiation. Recent studies have shown that mechano-transduction cues greatly influences the generation and maintenance of CSCs and eventually metastasis (Chen and Kumar, 2017). In addition, mechanical properties such as deformability and adhesiveness are different for CSCs as compared to the bulk tumor population (Saliba et al., 2010; Zhang et al., 2012; Babahosseini et al., 2014; Chen J. et al., 2016). These advances have enabled attempts to isolate and identify CSCs based on biophysical marker using engineering techniques such as microfluidic devices (Saliba et al., 2010; Gossett et al., 2012; Zhang et al., 2012; Liu et al., 2015; Chen J. et al., 2016) in a label-free manner.

For instance, CSCs were enriched based on their adhesive traits using a microfluid chip having micro-channels coated with basement membrane extract. The cells entered into the chip driven by hydrodynamic forces. While highly adhesive cells were captured in micro-channels, less adhesive cells were collected from the outlet, which were shown to be enriched in CSCs, had greater motility and were resistant to chemotherapeutic drugs (Zhang et al., 2015). This study emphasizes the interconnections between EMT, stemness, and drug resistance (Jolly et al., 2019), and the use of microfluidics in investigating these associations (Nath et al., 2019). Similarly, another microfluidic device fitted with microbarriers was used to isolate cancer cells based on their deformability *in vitro*. The more deformable flexible phenotype was associated with expression of many genes involved in motility, metastasis and greater mammosphere formation efficiency (Zhang et al., 2012). Consistently, *in vivo*, deformability has been shown to be crucial for efficient extravasation of tumor-repopulating cells during metastasis as seen in zebrafish models (Chen J. et al., 2016; Figure 3).

Similar to biochemical heterogeneity observed within CSCs (Bocci et al., 2019; Moreira et al., 2019), CSCs can be biophysically strikingly different too (Table 2). In a recent study, Chen et al. (2019) showed the association of biophysical properties of CSCs and their ability to invade, migrate, and initiate tumors, using the SUM149 inflammatory breast cancer (IBC) stem cells. In this study, the authors sorted SUM149 CSCs based on the expression of aldehyde dehydrogenase enzyme (ALDH) and assayed biophysical properties of ALDH+ and ALDH– cells in terms of deformability, adhesion strength and contractility. ALDH+ cells displayed greater deformability, lower adhesion strength and reduced contractility relative to ALDH– cells, and resulted into enhanced functional phenotypes *in vitro* and greater tumor development *in vivo*. In addition, the authors isolated IBC cells based on their adhesive property using a microfluidic device and showed that the less adhesive cell population was ALDH– enriched, displayed enhanced *in vitro* invasion and migration as well as increased *in vivo* tumor development. Further, exogenous alteration of cell stiffness also resulted in changes in metastatic potential of these cells with less stiff cells showing greater invasion and migration (Chen et al., 2019). The results observed in this study corroborated well with earlier studies showing that cancer cells with greater deformability, lower adhesion strength and lower contractile force show enhanced metastatic potential (Swaminathan et al., 2011; Kraning-Rush et al., 2012; Byun et al., 2013; Sun et al., 2016; Bongiorno et al., 2018).

Therefore, with increasingly detailed characterization of biomechanical properties of various subpopulations of cancer cells and ECM, a “mechanosome” or “matrisome” signature may be helpful in identifying and isolating the most aggressive cancer cell subpopulations (Roy Choudhury et al., 2019).

## Single-Cell Methods to Identify CSCs and Their Subsets

Much of our current understanding about CSCs comes from studies performed on bulk cancer cell populations. However, bulk analysis masks the underlying intra-tumor heterogeneity and does not inform much about rare cell subpopulations within the tumor (Navin, 2015; Bhatia et al., 2019). Therefore, it becomes extremely critical to study the cancer cells at a single-cell resolution to better comprehend the CSC heterogeneity and plasticity (Etzrodt et al., 2014). Flow cytometry and Fluorescence-Activated Cell Sorting (FACS) is a widely used method for the isolation and characterization of single CSCs (Greve et al., 2012; Etzrodt et al., 2014). Also, lineage tracing can be used to follow the fate of individual cells (Figure 3; McKenna and Gagnon, 2019).

A recent study integrated FACS analysis of CSC markers with functional assays such as proliferation, self-renewal and multipotency tests, and observed that glioblastoma stem cell heterogeneity results from tumor plasticity which is determined by the microenvironment cues (Dirkse et al., 2019). Lineage tracing methods have also been utilized by researchers to decipher the properties of CSCs and has huge potential in understanding the transition of cellular states. Using intra-vital *in vivo* lineage tracing method in a genetic mouse model of breast cancer, Zomer et al. (2013) demonstrated the existence of CSCs in unperturbed mammary tumor. They also found that CSC state is plastic and can be activated, lost or deactivated. In another study, lineage tracing and transcriptional analysis of Notch1 expressing cells of intestinal tumors has led to the identification of a previously uncharacterized and undifferentiated stem cell population that contribute to tumor progression and heterogeneity (Mourao et al., 2019).

The advent of single-cell omics approaches has revolutionized our knowledge on CSC biology. Single cell genomic and transcriptomic analyses have provided invaluable insights regarding intra-tumor heterogeneity and clonal evolution in different cancers (Patel et al., 2014; Eirew et al., 2015; Hou et al., 2016; Puram et al., 2017). In addition to discerning the dynamics of clonal evolution, single cell omics methods have also enabled the identification of transitioning CSCs and their contribution to drug resistance (Puram et al., 2018; Sharma et al., 2018). Considering the rarity of CSCs, combining single cell transcriptomics with enrichment strategies such as flow cytometry or sphere assays are capable of drastically improving the characterization of CSC (Akrap et al., 2016; Jonasson et al., 2019). Single cell multi-omics approaches involving obtaining information from multiple components within a single cell is also gaining interest, because it facilitates the assessment of genotype and phenotype relationship in regulating the individual cell states (Macaulay et al., 2017; Liu et al., 2019).

Nonetheless, most of the single-cell omics analyses does not preserve spatial information as it requires the cells to be isolated from their microenvironment (Yuan et al., 2017). Another limitation is that a snap-shot analysis is inadequate to evaluate the dynamic nature of cellular processes (Skylaki et al., 2016). Transcriptomic profiling of cells using fluorescence *in situ* hybridization or sequencing will enable the decoding of the spatial regulation of cellular heterogeneity at single-cell resolution (Lee et al., 2014; Chen et al., 2015; Suva and Tirosh, 2019). Single-cell RNA sequencing data can be mapped to spatial transcriptomic data using advanced computational methods (Satija et al., 2015; Edsgard et al., 2018). Using a newly developed high-throughput automated single cell image analysis (HASCIA), the spatio-temporal factors regulating glioblastoma stem cell state transitions has been recently investigated (Chumakova et al., 2019). Integrating the transcriptomic and spatial data can significantly improve the interpretation of the CSC plasticity (Satija et al., 2015; Yuan et al., 2017). A recent work coupling large scale single cell-resolution 3D imaging strategy, lineage tracing and RNA sequencing in pTEN/Trp53 deficient mice models, observed extensive molecular heterogeneity and clonal plasticity within tumors and found that EMT is not a rare event within the tumors (Rios et al., 2019). Live single-cell imaging techniques are also being developed which overcomes the limitation of static snap-shot analyses in studying the temporal regulation of cellular state changes (Fumagalli et al., 2019). The number of genes analyzed by such studies are much less than snap-shot studies and the lack of specialized tools and computational methods for handling the large amount of data generated through such studies is a major challenge (Skylaki et al., 2016).

With emerging evidence about the potential of single-cell analysis in understanding the biology of CSCs, development of newer tools and analysis methods and integrative approach are required for better comprehending the cell state transitions and improved therapeutic strategies.

## Computational Methods to Identify CSCs

With the deluge of preclinical and clinical data being generated at a high-dimensional level, computational approaches to extract meaningful information and generate testable hypotheses are becoming more common (Suhail et al., 2019). Various “top–down” and “bottom-up” computational methods provide a framework to unravel novel insights into various aspects of the dynamics of cancer progression such as role of intra-tumoral heterogeneity, dynamics of EMT, CSCs and its role in metastases, evolutionary dynamics of cancer initiation and progression, prediction of treatment response and therapy resistance (Figure 3). While “top–down” methods use high-dimensional data and apply an inferential metric to identify patterns through machine learning and/or network reconstruction, the “bottom-up” approaches aim to elucidate the emergent dynamics of a phenomenon based on its mechanism-based description through mathematical modeling. Both approaches can be synergistically used to predict and/or interpret cellular behavior (Altrock et al., 2015; Jolly et al., 2017). Mechanism-based, i.e., “bottom-up,” mathematical models have been useful in understanding the dynamics of complex regulatory networks that modulate cancer stem cell behavior such as stem cell state transitions and dedifferentiation (Sehl and Wicha, 2018). Recent studies using mathematical models have predicted that cells in one or more hybrid E/M phenotypes are associated more with stemness as compared to cells in purely mesenchymal or purely epithelial (Jolly et al., 2014). These predictions have since been validated *in vitro* and *in vivo* (Grosse-Wilde et al., 2015; Bierie et al., 2017; Pastushenko et al., 2018; Kroger et al., 2019) and has been supported by clinical data (Jolly et al., 2019). How are the pathways of EMT and stemness interconnected with each other? These questions can be addressed by investigating *in silico* the coupling between core regulatory circuits of EMT, CSCs and other connected signaling pathways such as Notch signaling; this model predicted that altering the coupling strength between EMT and CSC networks and/or modulating Notch signaling can change the position of “stemness window” on the “EMT axis,” thus generating various subsets of CSCs in terms of EMT phenotypes (Bocci et al., 2018). Such CSC heterogeneity has been extensively seen across cancers (Liu et al., 2014; Giraddi et al., 2018; Zheng et al., 2018). A common unifying principle that has emerged upon investigating the regulatory networks underlying EMT, CSCs and related traits such as drug resistance has been the role of interconnected feedback loops in enabling multiple phenotypes (i.e., heterogeneity) and the ability to switch among them (i.e., plasticity) (Mooney et al., 2017; Tripathi et al., 2017; Jolly et al., 2018c). Phenotypic plasticity can abet the generation and maintenance of phenotypic heterogeneity (Celia-Terrassa et al., 2018; Hari et al., 2019); thus, breaking these feedback loops can be thought of as a novel potential therapeutic strategy to restrict phenotypic plasticity and/or heterogeneity (Celia-Terrassa et al., 2018; Hari et al., 2019).

“Data-based” models have also been valuable in decoding CSC signatures. A stemness index was derived using one-class logistic regression and observed to be higher in the metastatic breast cancer cells compared with primary tumors (Malta et al., 2018), suggesting the possibility of a signature specific to metastatic CSCs. Similar logistic regression models have been used to quantify the extent of EMT (George et al., 2017) that has revealed the heterogeneity of EMT phenotypes in various CSCs and their subsets (Bocci et al., 2018), hence strengthening the insights from “mechanism-based” or “bottom-up” models.

Another set of questions where mathematical models have been useful is estimating the fraction of CSCs in a tumor. Many studies have used population-level models to understand the difference in growth kinetics of CSCs and non-CSCs, and used that to offer a potential mechanistic underpinning of “tumor growth paradox,” i.e., accelerated tumor growth with increased cell death (Hillen et al., 2013). Typically, CSC represent a minor cell population within a tumor, major population being the non-CSCs which compete with the CSCs for space and resources. While induction of cell death results into death of bulk of non-CSCs which facilitates increase in CSC division, ultimately resulting into expansion of CSC population and increase in tumor progression (Hillen et al., 2013). In contrary, another study showed that the CSC population within a tumor is homeostatically maintained such that reducing CSC population below a threshold triggers extensive phenotypic switching of non-CSC to CSC population (Sellerio et al., 2015). Thus, while the dynamics and mechanisms of CSC generation, plasticity and maintenance remain to be comprehensively understood (Enderling, 2015), integrating these different modeling approaches with one another and with experimental and clinical data shall contribute to revealing this complex behavior at an intracellular and at a population level. Such an improved dynamic understanding can help identify optimal treatment strategies to reduce tumor burden, such as a combination of radiation and differentiation therapies (Bachman and Hillen, 2013), or a sequential treatment of drugs to tackle the *de novo* generation of CSCs (Gupta et al., 2011) and their functional attributes (Goldman et al., 2015).

## Spatial Organization of CSCs

Spatial heterogeneity is a fundamental feature of TME that contains diverse cell types (Yuan, 2016; Yuan et al., 2017). One canonical representation of spatial heterogeneity is the co-occurrence of vascular and hypoxic regions, as observed in solid tumors (Alfarouk et al., 2013). This heterogeneity can alter cellular phenotypes, for instance, glioblastoma cells in hypoxic regions have been shown to over-express epidermal growth factor receptor (EGFR) while those over-expressing platelet-derived growth factor receptor (PDGFRA) were enriched in vascular regions (Little et al., 2012). Similarly, hypoxic TME induced by anti-angiogenic agents can increase breast CSCs (Conley et al., 2012). Hypoxia can be acute, chronic or cyclic (intermittent), each with its unique effects on tumor progression (Saxena and Jolly, 2019). Thus, spatial heterogeneity of TME can give rise to differential spatial organization of cancer sub-populations within a tumor.

Varying levels of nuclear β-catenin expression was observed in different sub-populations of well-differentiated colorectal cancer, suggested to be regulated by TME (Brabletz et al., 2001). Cells in the invasive front of the primary tumor as well as metastases expressed high levels of nuclear β-catenin, and lacked the expression of membranous E-cadherin, indicative of an EMT. On the other hand, centrally located cells in the primary tumor and metastases showed cytoplasmic β-catenin and membranous E-cadherin expression, perhaps due to a mesenchymal-epithelial transition (MET) (Brabletz et al., 2001). Spatial heterogeneity with respect to EMT has been reported since in primary tumor (Jung et al., 2001; Schmalhofer et al., 2009; Bronsert et al., 2014; Liu et al., 2014; Grigore et al., 2016). Consistently, CD24^–^CD44^+^ mesenchymal breast CSCs were found in the invasive edge of the tumor, while the more epithelial or hybrid E/M CSCs, identified by ALDH1+, were localized in the interior regions of the tumor (Liu et al., 2014). Put together, these observations beg the question of what mechanisms might underlie such patterning. Recent efforts including a mathematical modeling analysis revealed that in the presence of a gradient of TGF-β (EMT inducing) signal, cell-cell communication among tumor cells mediated via Notch-Jagged signaling can recapitulate the experimentally observed spatial organization of CSCs sub-populations with varying EMT phenotypes (Bocci et al., 2019). *In vitro* knock down of JAG1 in SUM149 human breast cancer cells significantly reduced their tumor organoid formation, confirming the role of Notch-Jagged signaling in tumor progression (Bocci et al., 2019). Future studies can focus on gaining a understanding of other interconnected aspects of heterogeneity in TME.

Spatial heterogeneity of tumors can be used as a predictor of cancer prognosis and treatment response across different cancer types (Yuan, 2016). For example, colorectal cancer patients with high density of tumor infiltrating lymphocytes responded to anti-cancer therapy (Gong et al., 2018). Spatial heterogeneity can also significantly impact the time to occurrence in cancer cells exposed to continuous as well as adaptive therapies (Gallaher et al., 2018). Thus, the spatiotemporal dynamics of phenotypic changes induced by TME can be pivotal in aggravating aspects of tumor progression.

## Metabolic Plasticity of CSCs

Ever since Otto Warburg’s observation that cancer cells, unlike normal cells preferentially rely on glycolysis for energy production even under aerobic conditions, referred to as aerobic glycolysis or Warburg effect (Warburg et al., 1927), metabolic adaptation of cancer has been under extensive investigation (Liberti and Locasale, 2016; Peiris-Pages et al., 2016). To meet the varying metabolic demands set by the microenvironment during the course of tumor progression and to survive, cancer cells must dynamically rewire their metabolic phenotype and has been recognized as a hallmark feature of cancer (Hanahan and Weinberg, 2011). This ability to adapt is critical for tumor growth, metastasis and response to therapy (Dupuy et al., 2015; Luo and Wicha, 2015). Metabolic plasticity, also contributes to the tumor heterogeneity (Lehuede et al., 2016; De Francesco et al., 2018). Emerging evidence suggest that CSCs display extensive metabolic plasticity and can reprogramme their metabolism in a context dependent manner in response to the cues from the microenvironment (Albini et al., 2015; Sancho et al., 2016). Also, non-CSCs can acquire a stem-like character through changing the metabolic phenotype. This acquired stemness by altering metabolism is an emerging hallmark of cancer, known as “metabostemness” and contributes to the CSC plasticity (Menendez and Alarcon, 2014; De Francesco et al., 2018). There is no consensus regarding the metabolic phenotype of CSCs (Peiris-Pages et al., 2016). Although many reports show that CSCs are more glycolytic than the other differentiated cells, several other conflicting reports suggest that they prefer mitochondrial oxidative phosphorylation (OXPHOS). For example, CSC from the breast, lung, and colon cancers have been found to show higher glycolytic activity than the other cells (Ciavardelli et al., 2014; Liu et al., 2014). On the other hand, CSCs from pancreatic cancer and leukemia depend on OXPHOS for energy production (Skrtic et al., 2011; Lonardo et al., 2013; Sancho et al., 2015). Also, studies have shown that CSCs could switch from one metabolic state to another in response to various challenges microenvironment like pH, hypoxia and nutritional status (Singh et al., 2004; Sancho et al., 2015; Chae and Kim, 2018). This apparent discrepancy in these observations could be due to multiple reasons. One reason could be the intrinsic differences between the metabolic phenotype of different cancer types from which they were derived (Kim and DeBerardinis, 2019). Another major reason could be the metabolic plasticity of CSCs, existence of multiple CSC subsets, each of which may have an increased proclivity to exhibit a particular metabolic phenotype, reminiscent of observations for varying degrees of stemness observed along the spectrum of EMT states (Gammon et al., 2013; Peiris-Pages et al., 2016; Bocci et al., 2018). The inherent limitation associated with the use of distinct markers/techniques for the isolation and characterization of CSCs in these studies can be another confounding factor (Sancho et al., 2016; Snyder et al., 2018; Martano et al., 2019). Also, the preference of metabolic states relies heavily on the microenvironmental conditions, differences in the nutrient availability at the primary and metastatic sites (Dupuy et al., 2015; Pascual et al., 2018) and the co-operation (Pavlides et al., 2009) or competition with the stromal cells (Chang et al., 2015). The cause for the differences in the metabolic states are also often attributed to alterations in the mitochondrial mass, mitochondrial dynamics, biogenesis and mitochondrial DNA content (Guha et al., 2014; De Luca et al., 2015; Farnie et al., 2015; De Francesco et al., 2018). Metabolic reprogramming can be orchestrated by a wide array of signaling pathways (Ito and Suda, 2014; Papa et al., 2019). For example, overexpression WNT1/FGF3 signaling in MCF7 resulted in increased stemness by increased mitochondrial mass and thereby increasing the mitochondrial respiration (Lamb et al., 2015). Thus, various different cell-intrinsic and cell-extrinsic factors may modulate the “metabostemness.”

In addition to switching between glycolytic and OXPHOS phenotype, CSCs have also been shown to coopt these two pathways and exist in a hybrid metabolic state as suggested by recent studies (Yu et al., 2017). So, it is imperative to use drugs that block both glycolysis and OXPHOS to target CSC plasticity and has been found to inhibit tumor growth and metastasis (Cheong et al., 2011; Peiris-Pages et al., 2016). For instance, combining fasting induced hypoglycemia (to reduce glycolysis) with metformin, an OXPHOS inhibitor has impaired the metabolic plasticity of cancer cells by regulating PP2A-GSK3β-MCL-1 Axis (Elgendy et al., 2019).

Although most studies have focused on the glucose metabolism, the involvement of other metabolic pathways like lipid and amino acid metabolism still needs to be investigated further. A better understanding of the metabolic phenotypes and plasticity of CSC is required for the effective elimination of these cells.

## Discussion

The issue of phenotypic plasticity presents a clear and present danger in the treatment of cancer patients. Accumulating evidence suggests that CSCs consist of different sub-populations that can interconvert among different states through intracellular and intercellular regulatory networks. Over expression of one or more transcription factors or activating trans-differentiation processes such as EMT and metabolic alterations can drive the switch among CSCs and non-CSCs as well as between different subsets of CSCs. These adaptive strategies adopted by cells must be taken into account while devising new therapeutic strategies in the clinic in order to target all populations effectively.

The challenges to addressing this issue are multifold. The ability to identify the plastic CSC population using markers has its inherent problems which are further confounded by each individual patient’s unique biochemical and biomechanical signatures. The general consensus is that drug resistance is achieved through a transition to a slow cycling state which is reversible once the stress is removed (De Angelis et al., 2019). Understanding how the cells switch from a slow cycling state and reenter the proliferative phase of the cell cycle will be key to targeting this population which contributes to minimal residual disease.

Using latest developments in computational and experimental methods will allow us to map the different states of tumor cells within multiple locations in a tumor, thus enabling a more comprehensive view of the genetic and non-genetic heterogeneity that exists within a cancer. Correlating such intratumoral heterogeneity with cellular phenotypes will be key to devising better therapeutic options in patients to ablate the tumor cells stably within a patient.

## Author Contributions

RN and MJ designed the review. AT, KS, RM, MJ and RN wrote, read, and approved of the manuscript.

## Conflict of Interest

The authors declare that the research was conducted in the absence of any commercial or financial relationships that could be construed as a potential conflict of interest.
